# Transcatheter arterial chemoembolization combined with radiofrequency ablation delays tumor progression and prolongs overall survival in patients with intermediate (BCLC B) hepatocellular carcinoma

**DOI:** 10.1186/1471-2407-14-849

**Published:** 2014-11-19

**Authors:** Xin Yin, Lan Zhang, Yan-Hong Wang, Bo-Heng Zhang, Yu-Hong Gan, Ning-Lin Ge, Yi Chen, Li-Xin Li, Zheng-Gang Ren

**Affiliations:** Liver Cancer Institute & Zhongshan Hospital, Fudan University, 136 Yi Xue Yuan Road, Shanghai, 200032 China; Key Laboratory of Carcinogenesis and Cancer Invasion, Ministry of Education, Shanghai, China

**Keywords:** Hepatocellular carcinoma, Transcatheter arterial chemoembolization, Radiofrequency ablation, Combination therapy, Survival

## Abstract

**Background:**

This study was designed to evaluate the effectiveness of radiofrequency ablation in patients with intermediate (BCLC B) stage hepatocellular carcinoma who underwent transcatheter arterial chemoembolization.

**Methods:**

Included in this study were 211 patients with intermediate stage HCC who underwent initial transcatheter arterial chemoembolization and were potentially amendable for radiofrequency ablation (single tumor with diameter 5-8 cm, median 6.0 cm; 2–5 multiple nodules with diameter less than 5 cm) between January 2005 and December 2011. According to the inclusion and exclusion criteria, 55 patients were treated with following radiofrequency ablation, and the remaining 156 patients were treated with transcatheter arterial chemoembolization alone. The treatment effectiveness, local tumor control and survival outcome between the two groups were compared.

**Results:**

The complete tumor necrosis rate after treatment was 76.9% in combination group *vs*. 46.5% in transcatheter arterial chemoembolization alone group (P = 0.02). The major complication rate was 1.8% in combination group *vs*. 2.6% in transcatheter arterial chemoembolization alone group. Follow-up observation showed that the total tumor control rate was 74.5% in combination group versus 54.5% in transcatheter arterial chemoembolization alone group (P < 0.001). The 1-, 3- and 5-year survival rates in combination group were significantly higher than those in TACE alone group (P = 0.01).

**Conclusions:**

Radiofrequency ablation following initial transcatheter arterial chemoembolization delays tumor progression and prolongs overall survival of patients with intermediate stage HCC tumors.

## Background

Hepatocellular carcinoma (HCC) is the 6^th^ most common cancer worldwide [1]. Surgical resection and liver transplantation are the current mainstays in the treatment of HCC patients. Unfortunately, only about 20% HCC patients are candidates for resection [2], and non-surgical therapy is the only option currently available for most patients with intermediate or advanced HCC.

According to the Barcelona Clinic Liver Cancer (BCLC) guideline, radiofrequency ablation (RFA) or surgical resection is only indicated for patients with early stage (BCLC stage A) HCC, and transcatheter arterial chemoembolization (TACE) is the first-line treatment for patients with intermediate (BCLC stage B) HCC [3, 4]. Although it has been reported [5–7] that chemotherapy combined with ischemic necrosis induced by arterial embolization could delay tumor progression and improve patient overall survival (OS), it is usually difficult to necrose the target lesion completely by TACE alone. Extracapsular or intrahepatic tumor invasion is likely to occur after TACE due to incomplete embolization. In addition, TACE may potentially cause hypoxia within the tumors, and ischemic injury after TACE could induce the up-regulation of vascular endothelial growth factor (VEGF) [8], which may favor HCC growth, invasion and metastasis. Therefore, new strategies are needed to improve the outcome of patients with intermediate HCC who undergo TACE treatment.

RFA has emerged as a new curative treatment owing to its safety and effectiveness for early-stage small HCC [9–12]. In comparison with TACE, the advantage of RFA is curative local control of small HCC, but it is less favorable for complete tumor necrosis of tumors larger than 5 cm [13]. The combination of TACE and RFA has several advantages over RFA or TACE alone. First, as a downstage treatment, TACE can reduce tumor burden, decrease viable tumor volume before RFA, thus increasing the ablation rates of large tumors. Second, after TACE or repeated TACE procedures, the main artery supplying the tumor may be narrowed or even be occluded, and snaking arterioles may be regenerated from the phrenic, intercostal, gastric and superior mesenteric arteries [14], making it difficult to selectively catheterize the feeding artery to control residual tumor cells. While subsequent RFA can directly ablate the refractory tumors. Third, it is generally believed that recurrences after curative treatment for HCC in the early post-treatment period arise, not because of incomplete treatment of the primary tumor but because of pre-existing microscopic tumor foci that are not detected by imaging modalities [15]. TACE can target undetected these satellite lesions surrounding the main tumor, label the range and size of the tumor, thus providing guidance for RFA [16]. TACE combined with RFA has been reported to be effective for local control of medium-sized HCC tumors (3-5 cm) [17]. However, whether such combination therapy could provide therapeutic benefits to intermediate HCC unsuitable for RFA monotherapy has not been clarified. The purpose of this study was to evaluate the effectiveness and survival benefit of the TACE+RFA approach to the management of intermediate HCC.

## Methods

### Patients and enrollment criteria

From January 2005 to December 2011, 747 patients with intermediate (BCLC B) HCC received first-line TACE treatment at the Liver Cancer Institute of Fudan University Zhongshan Hospital (Shanghai, China). The diagnosis of HCC was confirmed pathologically or clinically according to the AASLD criteria [18]. Before TACE treatment, patients were primarily evaluated by experienced surgeons and were excluded the possibility of liver resection or transplantation. After initial TACE treatment (1–5 sessions), 211 patients who were potential candidates for subsequent RFA were further evaluated and included into this study based on the inclusion criteria: 1) the presence of a single HCC tumor ≤8 cm in diameter, or multi-nodular HCC tumors (n ≤ 5) small than 5 cm in diameter before initial TACE; 2) the presence of viable residual HCC with retained iodized oil after TACE as shown by the follow-up liver CT and/or MRI scan;3) the absence of portal vein invasion and extrahepatic metastasis; and 4) Child-Pugh class A or B. Of the 211 patients, 55 patients received combined RFA treatment based on the following criteria: 1) viable residual tumors after TACE could be detected by follow-up ultrasonography 2) residual tumors could be possibly ablated with curative intention by RFA; 3) absence of severe coagulopathies, such as prothrombin time ≤16 s or platelet count >50000/mL; and 4) patients who signed informed consent for RFA. The other 156 patients who were not suitable for RFA and received repeated TACE treatment were assigned to TACE alone group based on the following reasons: 1) tumors were poorly visible on planning ultrasound; 2) percutaneous RFA was infeasible due to the high risk location of thermal injury or could result in incomplete ablation due to the inadequate electrode path; 3) there existed coagulopathies such as prothrombin time >16 s or platelet count <50000/mL; and 4) patients were unwilling to receive additional RFA treatment due to economic or other personal reasons, although their residual tumors could be treated with combined RFA. Treatment effectiveness, local tumor control and survival outcome of the patients between the two groups were compared retrospectively. The TACE or RFA treatment procedures were according to our institutional standard treatment protocol at Fudan University Zhongshan Hospital. Informed consent was obtained from all patients and the study protocol was complied with the ethical standards of the Helsinki Declaration. This study was approved by the Medical Ethics Committee of Fudan University Zhongshan Hospital.

### Transarterial chemoembolization

TACE was performed as previously described [19]. Briefly, after introduction of a 5 F (Cook©, Bloomington, USA) or 4 F RH catheter (Cordis ©, CA, USA) using the Seldinger technique through the femoral artery, an angiographic survey of the abdominal vessels was performed. Depending on the size, location and arterial supply of the tumor, the tip of the catheter was advanced toward the tumor-feeding arteries for selective embolization of all tumors. Segmental embolization was also performed in small tumors by using a microcatheter (Terumo©, Tokyo, Japan) if needed. Oxaliplatin (100–150 mg) and/or 5-fluorouracil (500–1000 mg) were infused. Epirubicin (30–60 mg) or Mitomycin C (5–10 mg) mixed with 5-30 ml lipiodol was carefully injected under the survey of fluorescence. In hypervascular tumors where embolization was insufficient, gelatin sponge particles or strips were used for further embolization. The dose of the chemotherapeutic agent and lipiodol and the quantity of embolic material were determined based on the tumor burden, vascularity and liver function reverse.

### Radiofrequency ablation

After TACE treatment, a dynamic contrast CT or MRI scan was performed to evaluate the post- TACE tumor response. Patients who were considered as potential candidates for RFA were further examined by ultrasound or contrast ultrasound. Lesions to be ablated should be clearly visible on ultrasonography with a safe path. Ablation procedures were conducted in all patients with curative intention. The details of the treatment procedure were the same as described in our previous study [20]. Generally, RFA was performed with the patients under local anesthesia and real-time ultrasonographic guidance by using the RITA system (RITA Medical Systems Inc., Mountain View, CA, n = 14) or Cool Tip system (Valleylab, Boulder, CO, USA, n = 41). Two cycles (12 min each) were required during RFA. Overlap ablation was allowed to cover the whole tumor nodule to achieve a sufficiently safe margin of 0.5-1 cm if possible.

### Treatment assessment and follow-up observation

After each session of TACE treatment or RFA, a contrast-enhanced CT or MRI scan in 1–2 months was performed to evaluate the tumor response and side effects. In patients with residual viable tumors or incomplete ablation in the remaining liver, an additional session of TACE or RFA was performed as appropriate. Patients who showed no evidence of viable tumors were followed up every 2–3 months for serum alpha-fetoprotein (AFP), abdomen ultrasonography and chest X-ray. For patients with test results suggestive of tumor recurrence, CT and/or MRI were used. Tumor response was evaluated according to the modified Response Evaluation Criteria in Solid Tumors [21]. Local tumor control was assessed by the tumor control rate at 6 month after treatment, and time to tumor progression, which was defined as the interval from the date of initial treatment to the date of tumor progression, death or the last follow-up visit. Overall survival was calculated from the date of entry into the treatment to the date of death or the last follow-up.

### Statistical analysis

Quantitative data were compared using the Student *t* test. Categorical data were analyzed by the chi-square test or Fisher exact test as appropriate. Survival analysis was estimated by the Kaplan-Meier survival method and compared by the log-rank test. Possible prognostic factors influencing tumor progression and overall survival were analyzed using a Cox proportional hazards regression model. Statistical significance was set at P < 0.05 (two sided). All analyses were performed with the software SPSS 13.0 for Windows (SPSS, Inc., Chicago, IL, USA).

## Results

### Patient characteristics

All patients were followed up after initial treatment until December 2012. The median follow-up period was 23 months (range 2–71 months).The patient characteristics of TACE+RFA group and TACE alone group are summarized in Table 1. There was no significant difference in gender (P = 0.56), age (P = 0.99), AFP level (P = 0.61), tumor size (P = 0.99), tumor number (P = 0.30) and Child-Pugh grade (P = 0.98) between the two groups.

**Table 1:** **Clinicopathalogical variables in TACE+RFA group and TACE alone group**

Variables	TACE	TACE+RFA	P value
	n = 156	n = 55	
Gender			
Male	138(88.5%)	47(85.4%)	0.56
Female	18(11.5%)	8(14.6%)	
Age (years)			
<=50	54(34.6%)	19(34.6%)	0.99
>50	102(65.4%)	36(66.4%)	
HBsAg			
Positive	118(75.6%)	36(65.5%)	0.14
Negative	38(24.4%)	19(34.5%)	
Child-Pugh grade			
A	136(87.2%)	48(87.3%)	0.98
B	20(12.8%)	7(12.7%)	
AFP (ug/L)			
Positive >20	108(69.2%)	36(65.5%)	0.61
Negative <=20	48(30.8%)	19(34.5%)	
Tumor number			
Single	115(73.7%)	35(66.2%)	0.30
Multiple	41(26.3%)	20(33.8%)	
*Tumor size			
Medial (range, cm)	6.0(5–8)	5.9(5–8)	0.99

AFP: α-fetoprotein.

HBsAg: hepatitis B surface antigen.

*Diameter of multiple tumors was calculated as the sum of the size of every single tumor.

### Technical effectiveness in TACE+RFA group

**Figure 1:**
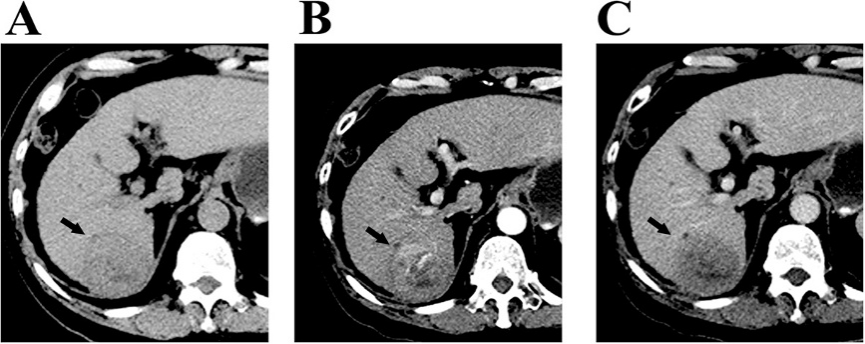
**Contrast-enhanced CT images obtained in a patient with 6-cm single HCC before TACE treatment. (A-C)** show a patient with hepatitis B-induced liver cirrhosis and a 6-cm solitary HCC tumor in the hepatic segment VI. The contrast-enhanced CT scan before TACE revealed arterial enhancement of the HCC lesion.

**Figure 2:**
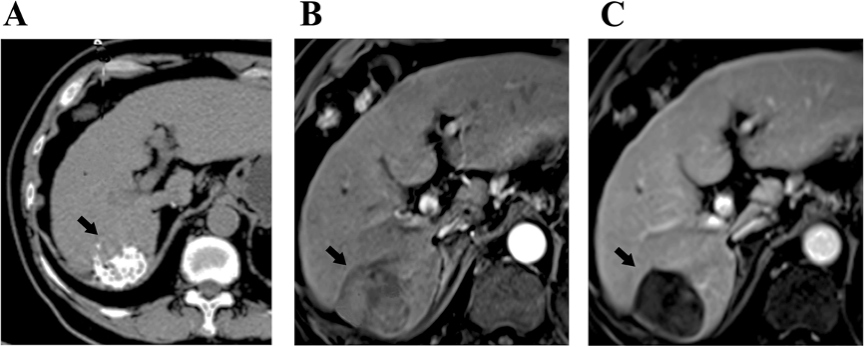
**Radiofrequency ablation after transarterial chemoembolization was performed on the same patient with 6-cm HCC after initial TACE treatment. (A)** CT scan after TACE treatment shows lipiodol uptake in the central aspect of the lesion. **(B)** Contrast-enhanced MRI scan at 4 weeks after RFA shows complete tumor necrosis without arterial enhancement within the lesion. **(C)** Contrast-enhanced MRI scan at 6 months after combination treatment shows no tumor recurrence in the liver.

In TACE+RFA group, patients received median 2.0 (range 1–5) sessions of TACE treatment, followed by1-3 sessions of RFA. While in TACE alone group, patients received median 3.0 (range: 1–9) sessions of TACE treatment. Due to the presence of residual enhanced lesions after treatment, 18 tumors in 9 patients received additional sessions of RFA or TACE in TACE+RFA group. The follow-up CT or MRI scan showed that 60 tumor nodules in RFA+TACE group (Figures 1 and 2) and 108 tumor nodules in TACE alone group were completely necrosed, resulting in a technical effectiveness rate of 76.9% (60/78) and 46.5% (108/232), respectively (P < 0.001).

### Safety evaluation

Most patients in TACE+RFA group experienced post-ablation syndrome, including fever, general fatigue and abdominal pain that persisted for 1-5days after RFA. The minor complications were asymptomatic self-limiting pleural effusion (1/55, 1.8%) and local thermal skin injury (1/55, 1.8%). A major complication was observed in one patient (1/55, 1.8%), who developed gastrointestinal bleeding within two week after RFA treatment. The most common adverse events in TACE alone group were abdominal pain, fever and nausea. However, these effects were transient and relieved within 1–2 weeks after TACE in most patients. Major complications included sever liver dysfunction (2/156) and upper gastrointestinal bleeding (2/156) within one week after TACE. The major complication rate was 2.6%.

### Local tumor control, tumor progression and associated risk factors

Using mRECIST criteria, the post-treatment tumor control rate was compared between the two groups. The tumor control rate was defined as the proportion of patients who achieved complete response (CR), partial response (PR), stable disease (SD) or progressive disease (PD) at 6 month after treatment. In TACE+RFA group, CR, PR, SD and PD were 60% (33/ 55), 10.9% (6/55) and 3.6% (2/55) and 25.5% (14/55) respectively versus 11.5% (18/156), 20.5% (32/156), 22.4% (35/156) and 45.5% (71/156) in TACE alone group. The total tumor control rate (CR+PR+SD) was 74.5% in TACE+RFA group versus 54.5% in TACE alone group (P < 0.001).

**Figure 3:**
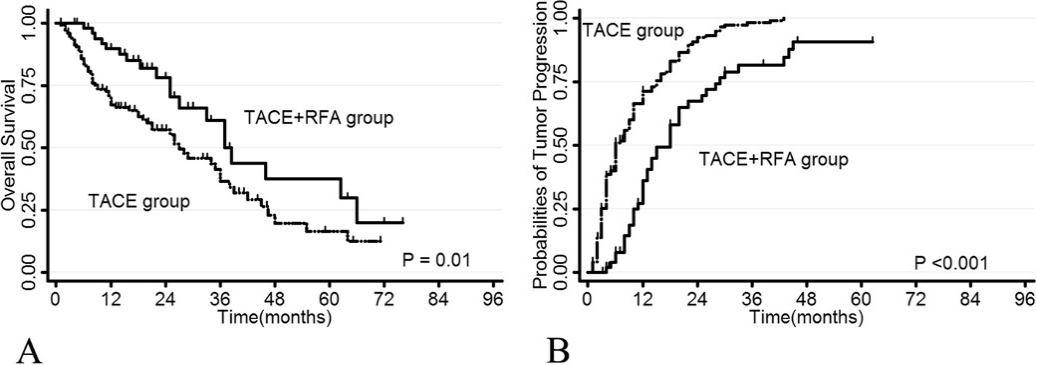
**Overall survival rate (A) and tumor progression rate (B) of patients in TACE+RFA group and TACE alone group.**

Follow-up observation showed that the median time for tumor progression was 6 months (range 1–28 months) in TACE alone group versus 13 months (range 3–46 months) in TACE+RFA group. The cumulative 1-, 3-, and 5-year tumor progression rates in TACE alone group were 71.4%, 98.3% and 100%, respectively, which were significantly higher than 36.0%, 81.6% and 90.8% in TACE+RFA group (P < 0.001; Figure 3B). Univariate analysis showed that single tumor and RFA treatment modality were correlated with decreased tumor progression (P = 0.04 and P < 0.001). Multivariate analysis with Cox proportional hazard model revealed that only RFA treatment was an independent factor associated with decreased HR for tumor progression (HR = 0.39, 95% CI: 0.27-0.56, P < 0.001) (Table 2).

**Table 2:** **Univariate and multivariate analyses of prognostic factors associated with tumor progression and overall survival**

Clinicopathological characteristics	TTP				OS			
	Univariate	Multivariate			Univariate	Multivariate		
	P	P value	HR	95% CI	P	P value	HR 95% CI	95% CI
Gender (female vs. male)	0.37	NS			0.30	NS		
Age, years (<50 vs. ≥50)	0.92	NS			0.18	NS		
HBV (no vs. yes)	0.17	NS			0.52	NS		
AFP ug/L (≤20 vs. >20)	0.11	NS			0.44	NS		
Child-Pugh grade (A vs. B)	0.23	NS			0.04	NS		
Tumor number (single vs. multiple)	0.04	NS			0.71	NS		
RFA treatment	<0.001	<0.001	0.39	0.27-0.56	0.01	0.01	0.51	0.31-0.86

AFP: alpha-fetoprotein; HBV: hepatitis B virus; CI: confidential interval; HR: hazard ratio.

NS: not significant; OS: overall survival; TTP: time to progression; RFA: radiofrenquency ablation.

### Overall survival

The survival rate in patients who received the combination treatment was significantly higher than that in patients who received TACE alone (Figure 3A). The post-treatment 1-, 3- and 5-year cumulative overall survival was 89.8%, 61.1% and 37.4% respectively in TACE+RFA group versus 67.2%, 36.6% and 16.5% in TACE alone group. (P = 0.01). The median survival time was 19 months (range 4–66 months) in TACE+RFA versus 11 months (range 2–47 months) in TACE alone group. Univariate analysis showed that RFA treatment (P = 0.01), along with Child-Pugh grade (P = 0.04) displayed relevance to overall survival. Multivariate analysis with Cox proportional hazard model showed that RFA treatment (HR = 0.51, 95% CI: 0.31-0.86, P = 0.01) was the only independent factor for long-term survival (Table 2).

### Subgroup analysis for stratification with tumor size and number

The present study included HCC patients either with a single tumor (size: 5-8 cm) or with multiple tumors (n ≤ 5, size ≤5 cm). Knowing that tumor size or tumor number is an important factor of local tumor control in RFA therapy and different tumor size or tumor number may result in differences in local tumor control and affect survival of HCC patients, we therefore analyzed the differences in tumor control and overall survival between the two subgroups (single-tumor group *vs*. multi-tumor group). Although the local control rate of the multi-tumor group was slightly higher than that of the single-tumor group (15/20, 75.0% *vs*. 26/35, 74.3%), the difference did not reach statistical significance (P = 0.51). Likewise, no significant difference in 1-, 3-, and 5-year tumor progression rate was observed between the single-tumor group and multi-tumor group (33.9%, 83.7% and 89.1% *vs*. 38.7%, 77.7% and 91.6%; P = 0.75), nor was there significant difference in overall survival (89.7%, 59.8% and 36.0% *vs*. 89.5%, 63.8% and 38.2%; P = 0.57).

## Discussion

The combined use of TACE and RFA is a common practice in the treatment of small HCC tumors. However, whether this combination approach is more effective than TACE alone in the treatment of patients with intermediate stage HCC is unclear. In the present study, we compared the treatment benefits between TACE+RFA and TACE alone in 211 patients diagnosed with intermediate stage HCC potentially amenable to RFA (single nodule with diameter 5-8 cm; 2 ~ 5 multiple nodules with diameter less than 5 cm). The results indicate TACE combined with RFA delays tumor progression and prolongs overall survival in patients with intermediate stage HCC.

The therapeutic effect of TACE+RFA has been described in several reports. Buscarini et al. [22] reported that the combined use of RFA and TACE increased the volume of coagulation necrosis in 14 HCC patients (mean diameter 5.2 cm). In a multicenter clinical trial, Lencioni et al. [23] reported successful ablation of HCC tumors (range 3.5–8.5 cm) in 51 (82%) of the 62 HCC patients treated with TACE and RFA. In accordance with these previous studies, we reported a successful ablation rate of 76.9% in BCLC stage B HCC lesions. Not a few studies [24, 25] have demonstrated that combined use of RFA and TACE is safe, with a relatively low major complication rate. Similar to previous studies, we also found that the patients were able to tolerate TACE+RFA treatment with no death-related event occurring in our group of HCC patients. The major complication rate was low in both TACE+RFA and TACE alone groups (1.6% *vs*. 2.8%).

According to the consensus of RFA guideline, RFA is generally considered as an alternative treatment to partial hepatectomy for early small HCC tumors (≤5 cm), especially for patients with impaired liver function. Without combination therapy, RFA alone should not be indicated in HCC tumors larger than 5 cm, because it is difficult for RFA alone to achieve complete ablation and favorable local tumor control in large tumors [26]. However, serving as a downstage treatment before RFA, TACE can reduce tumor burden by chemoembolization and increase the ablation rates of large tumors in a combination therapy. It also targets undetected satellite lesions surrounding the main tumor, labels the range of tumor, provides guidance in RFA procedure, thus increasing the possibility of complete ablation of the main tumor as well as its surrounding satellite lesions. We therefore anticipated that TACE+RFA combination would provide a favorable local control in the treatment of BCLC stage B HCC. Indeed, we found that the total tumor control rate was 74.5% in TACE+RFA group, which is significantly higher than 54.5% in TACE alone group (P < 0.001). The cumulative 1-, 3- and 5-year tumor progression rate in TACE+RFA group was 36.0%, 81.6% and 90.8% respectively, which are significantly lower than 71.4%, 98.3% and 100% in TACE alone group (P < 0.001). Our subsequent subgroup analysis showed that combined RFA seemed to have the same effect on local tumor control in patients with either solitary tumor (size: 5-8 cm) or multiple tumors (n ≤ 5, size ≤5 cm). In addition, multivariate analysis showed that it was combined RFA treatment (HR = 0.51, 95% CI: 0.31-0.86, P = 0.01) rather the tumor size or tumor number that was the only independent factor for tumor progression. All these results indicate that the combination regime offers better efficacy of local thermal ablation and extends the indication of conventional RFA ablation with respect to the tumor size and number, thereby facilitating favorable local control of BCLC stage B HCC tumors.

It was also found in our study that the combination therapy significantly prolonged overall survival of patients with intermediate stage HCC. The postoperative 1-, 3- and 5-year cumulative OS was 89.8%, 61.1% and 37.4% respectively in RFA+TACE group versus 67.2%, 36.6% and 16.5% in TACE alone group. The median survival time of patients undergoing combination therapy was significantly longer than that of patients receiving TACE alone (19 months vs. 11 months, P = 0.01). These results are not surprising. TACE combined with RFA provides a better local control and a lower tumor progression rate as compared with TACE alone therapy, thus contributing to a favorable survival outcome. In addition, combined TACE and RFA could overcome tumor burdens more effectively in large HCC tumors and avoid repeated TACE treatments that may damage liver function, thus providing significant survival benefits.

As a regional interventional therapy, RFA has led to a major breakthrough in the management of HCC. Real-time virtual sonography and overlap procedures increase the number of patients eligible for RFA treatment, especially for large HCC tumors. However, when considering the treatment guideline for unresectable HCC, most patients would be excluded from radical treatment owing to non-early stage tumors. Thus, clinicians may not be able to avoid the selection of palliative TACE instead of RFA or operation. Our series has provided encouraging evidence of the efficacy of TACE and RFA combination in the treatment of intermediate stage HCC patients. We suggest that subsequent tumor ablation should be considered in patients with residual tumors that are potentially amenable to RFA after TACE treatment. In other words, patients with intermediate stage HCC should be examined by ultrasound or contrast ultrasound after TACE treatment to determine the possibility and feasibility of receiving subsequent RFA. For example, patients with single HCC nodules larger than 5 cm could be initially treated with TACE, and then with RFA when the tumor is shrunken and can be ablated by using RFA, since such combination therapy can confer a favorable prognosis compared with monotherapy.

Our study has several limitations. First, this study was retrospectively performed and was not randomized in design. Second, all planning RFA procedures were performed in a single institution. Therefore, the results obtained in this study might be influenced by both the experience of the physician and the patient population. Finally, 73% of our patients had a history of positive HBV, which differs greatly from studies in the United States, Europe, and Japan. Thus, the treatment modality needs further investigation in HCC patients from these areas.

## Conclusions

In summary, our study demonstrates that combination therapy with TACE and RFA is an effective and safe treatment that may delay tumor progression and prolong overall survival in patients with BCLC B hepatocellular carcinoma. On the basis of our findings, this newly developed combination therapy is likely to be a promising therapeutic option for intermediate stage HCC. Future prospective studies are warranted to further confirm the benefits of this combination therapy.
